# Weighty: NICE's Not-So-Nice Words

**DOI:** 10.3389/fpsyg.2016.01919

**Published:** 2016-12-06

**Authors:** Lorena Lozano-Sufrategui, Andrew C. Sparkes, Jim McKenna

**Affiliations:** School of Sport, Institute of Sport, Physical Activity and Leisure, Leeds Beckett UniversityLeeds, UK

**Keywords:** obesity, stigma, public health, weight management, terminology

## Introduction—NICE guidance

The National Institute for Health and Care Excellence (NICE) provides national guidance to improve health and social care in England. It currently influences bodies such as the NHS, local authorities, employers, and anyone else involved in delivering care or promoting well-being. NICE is also part and shapes the dominant discourse surrounding “obesity” in England. For example, in 2016, NICE published the new quality standard on the prevention of obesity and lifestyle weight management programmes (NICE, 2016). This policy builds on NICE's (2014) public health guideline PH53, which makes recommendations on the provision of weight management services for adults who are “overweight or obese *[sic]*” (NICE, 2014, p. 3). Both documents acclaim that weight management services cause no harm to individuals who participate in them. Specifically, NICE (2014) recommended that the providers of weight management programmes prevent weight stigma by being respectful with the terminology they use:

Be aware of the effort needed to lose weight, prevent weight gain or avoid any further weight gain. Also be aware of the stigma that adults who are overweight or obese *[sic]* may feel or experience. Ensure the tone and content of all communications is respectful and non-judgmental. In addition, the terminology used to describe someone's condition *[sic]* should respect how they like to be described.

(p. 4)

## NICE recommendations—self-defeating adverts?

Despite advising practitioners to be careful with the terminology they use, NICE recommendations use the stigmatizing biomedical labels “overweight” and “obese.” This suggests that NICE has overlooked the body of literature that documents the negative psychosocial impact these labels have on the people they aim to describe. Sociological research suggests that for people with “excess” weight, the term “obese” evokes stronger negative evaluations than the term “fat” (Vartanian, 2010). Similarly, according to Monaghan (2008, p. 39):

[O]besity might be a technically “neutral” term in biomedicine but it is a stigmatizing concept in everyday life. It is typically associated with physical extremes and the “Other,” such as the person who is seriously impaired because of his size.

Puhl et al. (2013) also demonstrated that in public preferences for weight-based terminology used by healthcare providers to describe higher-body weight, “obese” was rated as one of the most undesirable, stigmatizing, and blaming words. Some tentative solutions to this issue of “othering” by labeling includes the use of a type of language that considers the person holistically instead of defining them by a particular characteristic. In this field of research, this would mean using the terms “person with obesity” instead of “obese person” (Kyle and Puhl, 2014). However, critics of this approach suggest that this language has also failed to free the person from the adverse judgment associated to obesity (Meadows and Daníelsdóttir, 2016).

As a result, Vartanian and Smyth (2013) pointed out the paradox that the current situation generates. That is, public health campaigns should focus on facilitating behavior and on behavioral change, yet by unwittingly using stigmatizing labels they may impede the likelihood of behavior change for any given individual. In recent years, evidence has identified experienced (and internalized) stigma as a unique contributor to negative health outcomes and behaviors. Both correlational and randomized studies have concluded that adults and children who experience weight stigma are more likely to avoid exercise and physical activity, and to engage in unhealthy diets and sedentary behaviors (Bauer et al., 2004; Hayden-Wade et al., 2005; Schvey et al., 2011; Smyth and Heron, 2011). Furthermore, a recent systematic review by Puhl and Suh (2015) confirmed that weight stigma can reduce quality of life amongst individuals who experience it, interfering with their efforts to improve health, lose weight, or prevent weight gain.

Despite these negative consequences have been documented in social science research for decades (Puhl and Heuer, 2010), NICE has only recently attempted to acknowledge the public health implications of weight stigma (see NICE, 2014). Although this shows an improvement from previous guidelines, unfortunately NICE still fails to practice what it preaches: that is, in seeking to guide practitioners to “do good,” by using biomedical terms NICE is (un)consciously behaving in a manner that they advise “others” to avoid. For example, if their policy audiences (i.e., those who have daily, face to face contact with people with excess weight) adopt NICE's approach and use the terms “overweight” and “obese” in their practice, aren't practitioners most likely to *do* harm? This inconsistency is also evident in the recently released NICE (2016) quality standard, in which discussions around stigma and the terminology used to refer to people with “excess weight” are conspicuous by their absence.

## Exploring alternatives

Evidence suggests that the use of weight labels such as “overweight” or “obese” can negatively impact on a number of health behaviors (Essayli et al., 2016). Therefore, by using these terms NICE may be creating more problems than the ones they are trying to solve. For example, labeling individuals with “excess weight” or designing interventions aimed at “overweight and/or obese people” may counterproductively reduce the reach, adherence, and effectiveness of interventions. As a result, it makes sense to explore alternative approaches to health promotion for people with higher-body weights.

Moving beyond weight loss, a large body of research has shown that factors such as fitness, diet and lack of stigma are good predictors of health. For example, Barry et al. (2014) reviewed the fitness vs. fatness literature and found that fit “overweight” and “obese” individuals have similar mortality risk as their normal weight counterparts. Similarly, Matheson et al. (2012) reinforced the association between healthy lifestyle habits and decreased mortality risk regardless of body mass index. Moreover, Muennig et al. (2008) argued the potential role that stigma-induced stress can have in the pathophysiology of obesity, suggesting that social constructs of idealized body image can have harmful health effects. Even worse, Sutin et al. (2015) concluded that unfair treatment on the basis of body weight increases mortality risk.

As a result, there seems to be a consensus between researchers from different epistemological traditions to agree that the adoption of a weight-normative approach, which defines health on the basis of weight loss, is problematic (Tylka et al., 2014). Hence, it may be that the way forward to prevent stigma and promote health is to shift the focus to a weight-inclusive approach for health promotion, which emphasizes health and wellbeing as multifaceted and focuses on improving health access and preventing weight stigma (Bacon and Aphramor, 2011; Vartanian and Smyth, 2013; Tylka et al., 2014). Practically, this means that interventions that focus on fitness, diet and lack of stigma may be more effective—in terms of promoting individual's health—than the current weight loss model promoted by NICE. This point is supported by Tomiyama (2014), who found that any health improvements observed on weight loss programmes (e.g., changes in systolic blood pressure, fasting blood glucose, cholesterol, and triglyceride levels) have very little to do with weight loss *per-se*.

An example of such trans-disciplinary practice is Health at Every Size® (HAES)^1^, which aims to support improved health behaviors for people of all sizes without using weight as a mediator (Monteath and McCabe, 1997). This approach promotes physical activity that allows people of all sizes, abilities, and interests to engage in enjoyable movement, to the degree that they choose. HAES not only makes clinical sense, but it is also a more ethical, humane, and “nicer” approach to lifestyle improvement interventions than that suggested by NICE (2016).

Furthermore, Scambler (2009) suggested that interventions aiming to reduce stigmatization have to acknowledge social structure. Despite this, NICE remains obdurately biomedical and individualistic, and its attempts to empower individuals through a “top-down” approach seem incomplete. To address this limitation, it might be argued that greater consideration of non-biomedical literature, such as the evidence that has been discussed in this paper, is required in the NICE recommendations. Until then, its efforts to improve the health of the nation, including participation in diet and/or exercise interventions, are methodologically flawed, and may risk a counterproductive effect.

## Author contributions

All authors listed, have made substantial, direct and intellectual contribution to the work, and approved it for publication.

### Conflict of interest statement

The authors declare that the research was conducted in the absence of any commercial or financial relationships that could be construed as a potential conflict of interest.

## Footnotes

^1^Health At Every Size and HAES are registered trademarks of the Association for Size Diversity and Health and used with permission.
